# THE EFFECTS OF INDY ON FLY HEALTH

**DOI:** 10.1093/geroni/igz038.367

**Published:** 2019-11-08

**Authors:** Blanka Rogina, Pooja Patel, Jacob Macro, Michael Li, Ryan Rogers

**Affiliations:** 1 University of Connecticut Health Center, Farmington, Connecticut, United States; 2 Wentworth Institute of Technology, Boston, Massachusetts, United States

## Abstract

Indy (I’m not dead yet) gene encodes a plasma membrane transporter of Krebs’ cycle intermediates with highest affinity for citrate. Indy is the fly homolog of a mammalian mIndy (SLC13A5), which has the same physiological function. Reduced expression of the Indy gene extends longevity in fruit flies and worms. Genetic and pharmacological INDY reduction affects metabolism in flies, worms, mice, rats and non-human primates by affecting the levels of cytoplasmic citrate. In flies, INDY is predominantly expressed in the midgut, fat body and oenocytes, all tissues with a key role in metabolism. Our first goal was to examine our working hypothesis that INDY reduction in the midgut regulates citrate levels leading to metabolic changes that preserve intestinal stem cell (ISCs) homeostasis and slows aging by modifying Insulin/Insulin-like signaling (IIS). ISC homeostasis is vital for midgut homeostasis and contributes to health and longevity. We found that reduction of Indy preserves ISC homeostasis and intestinal integrity. The IIS is a key nutrient sensing pathway, which regulates growth, metabolism and longevity. Indy reduction is associated with decreased IIS activity. Our second goal was to examine the role of IIS in Indy mediated changes in ISC homeostasis and health. We found that at least some of INDY’s beneficial effects on fly health are mediated by the IIS.

